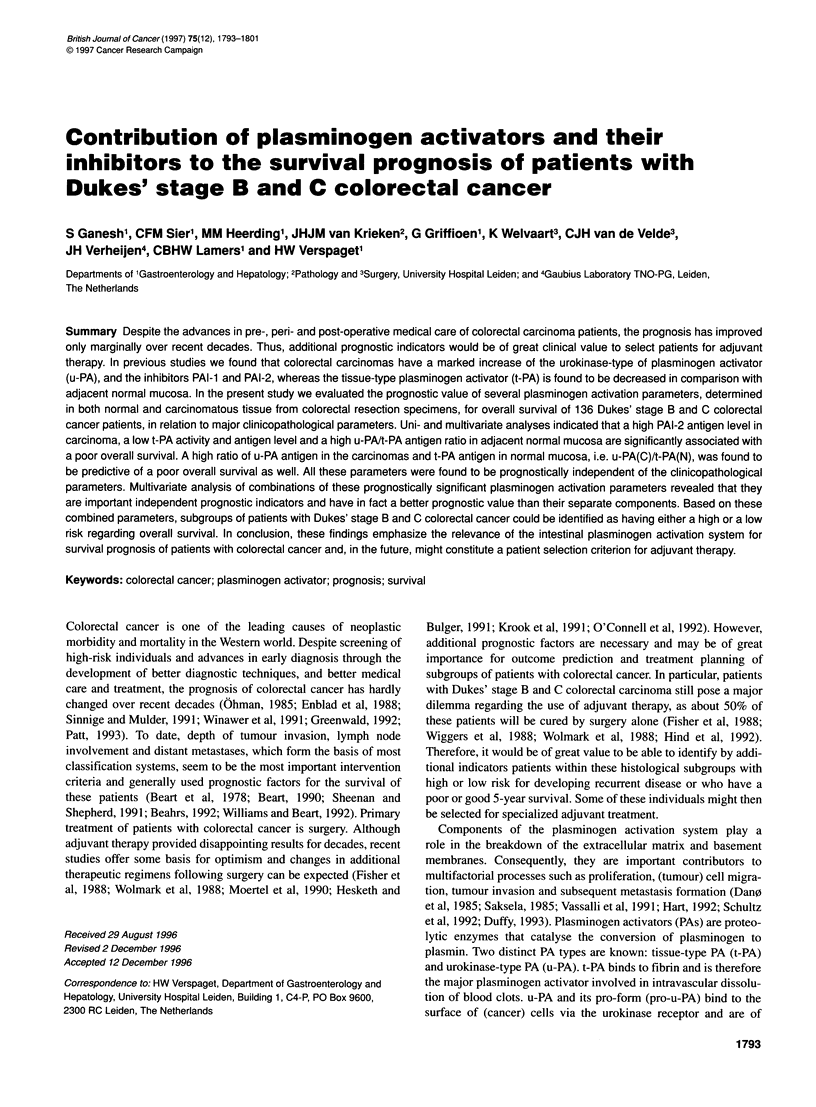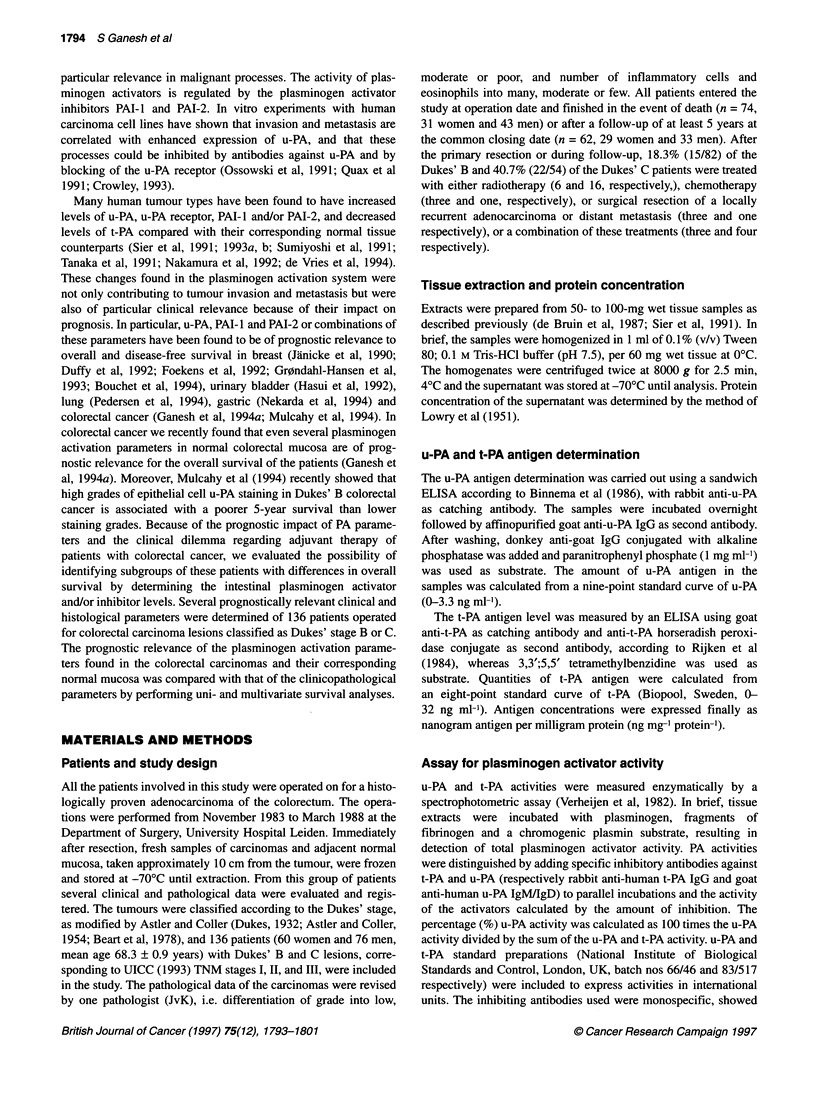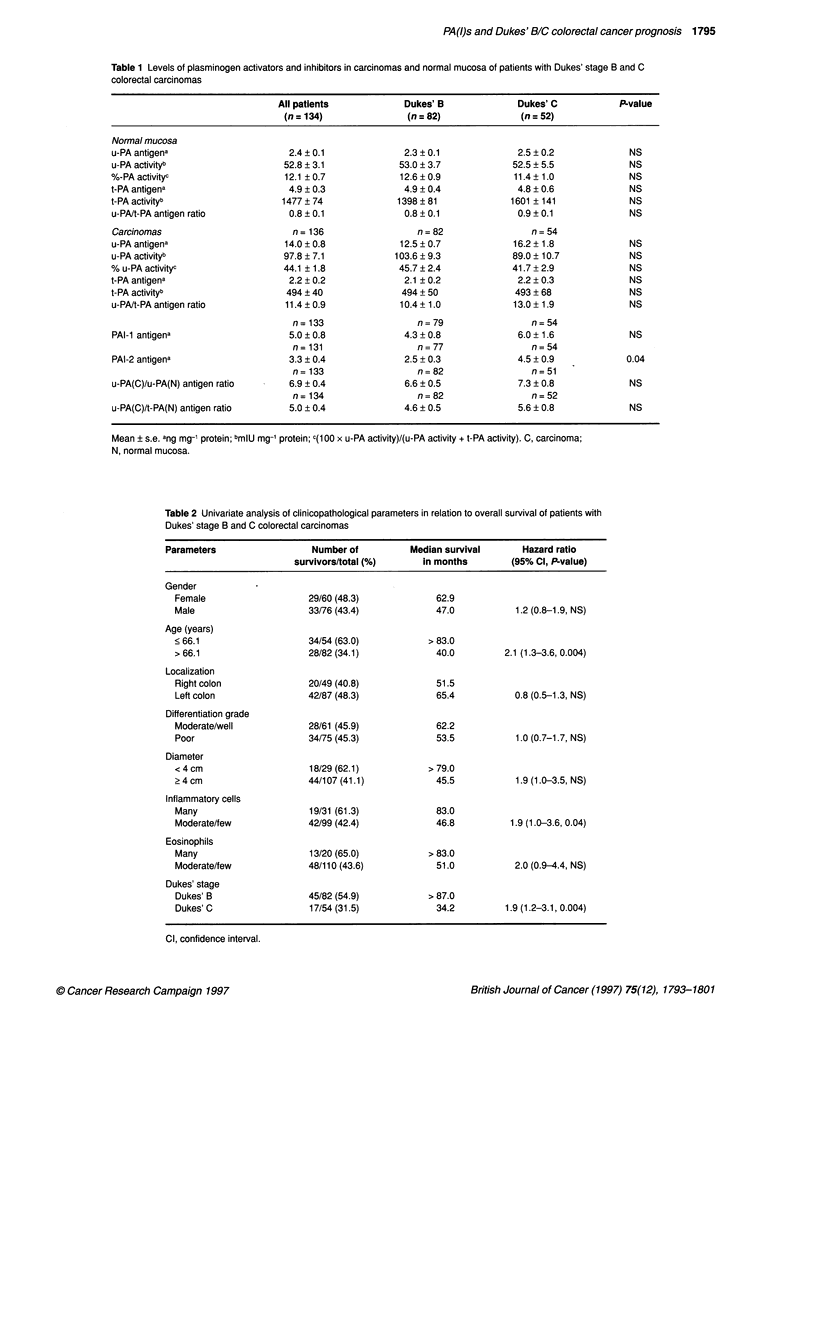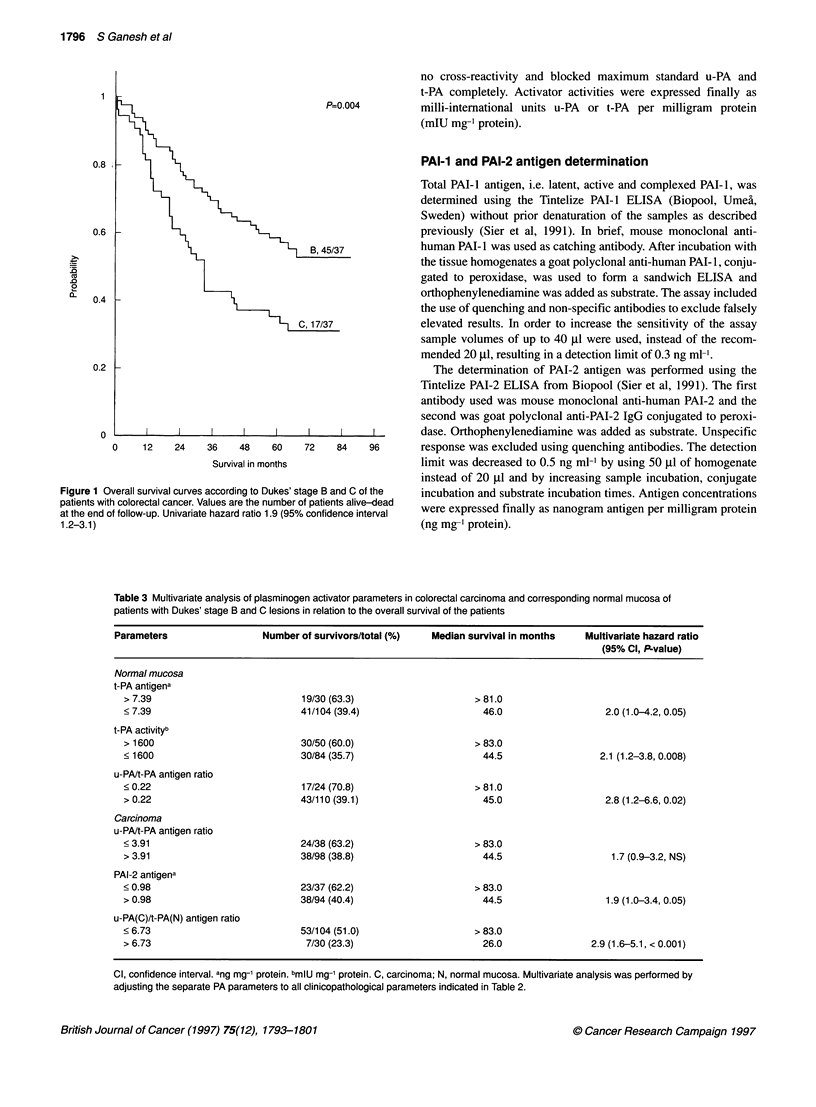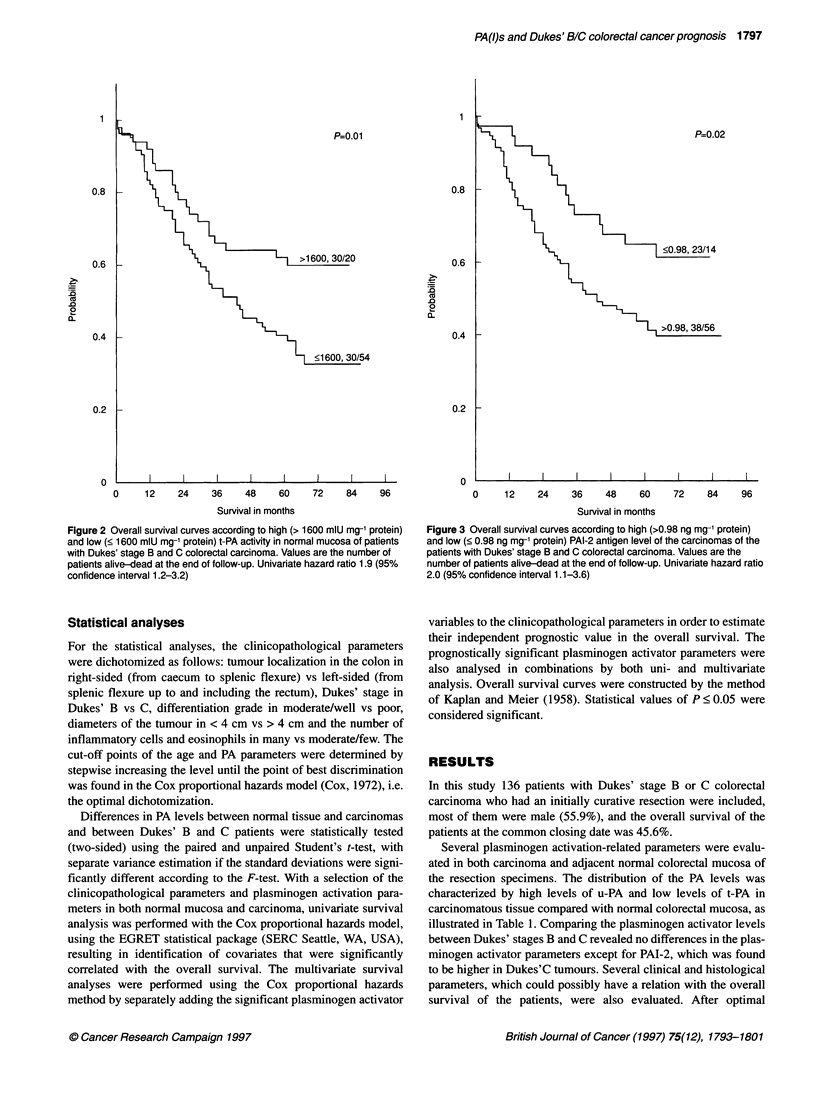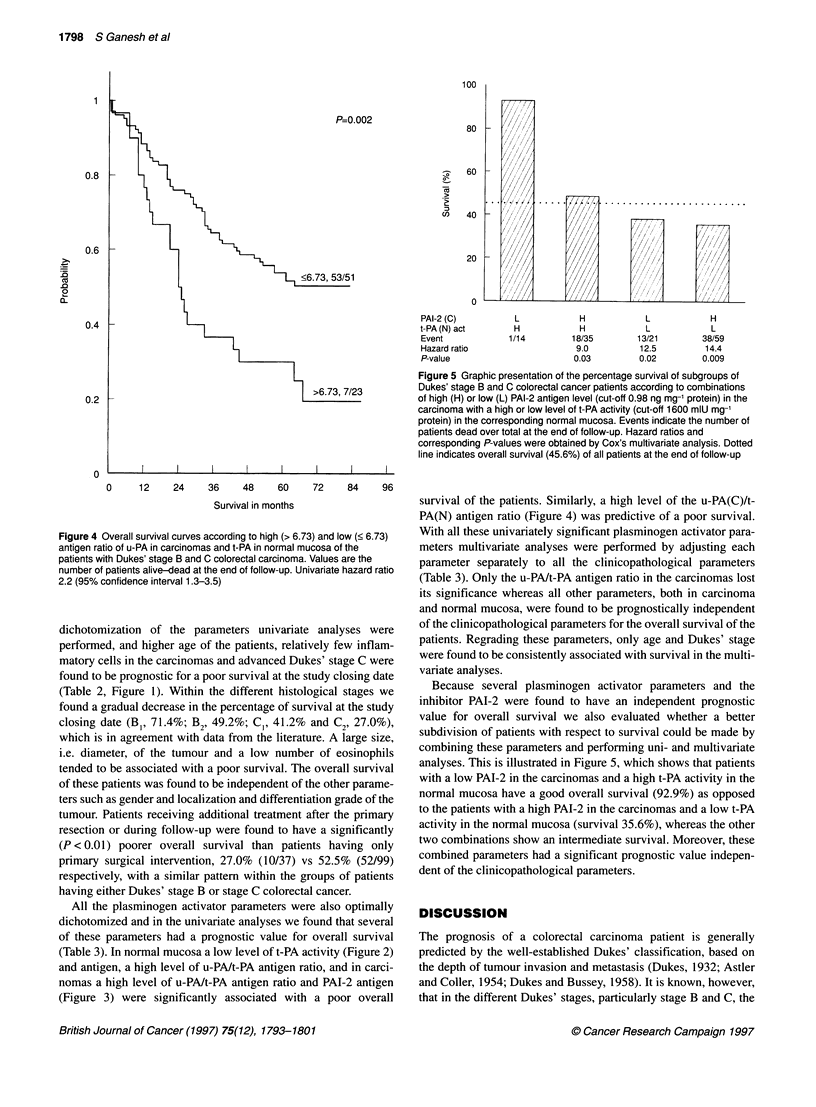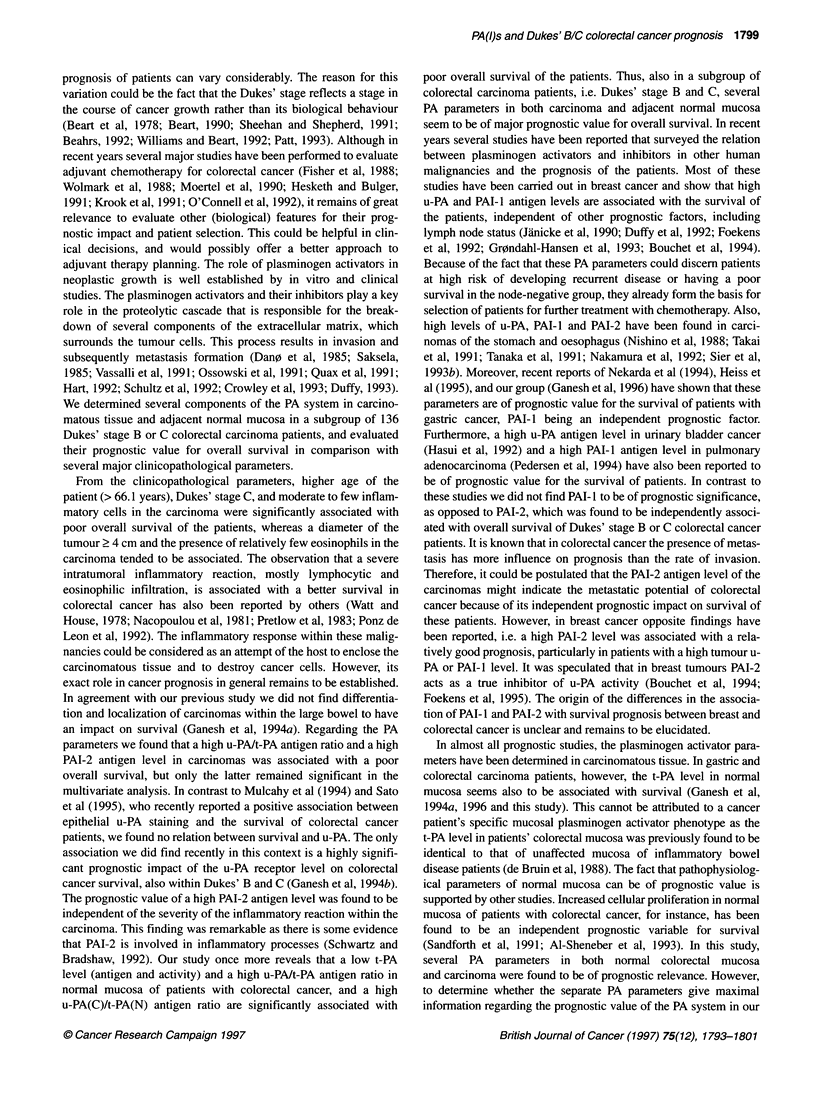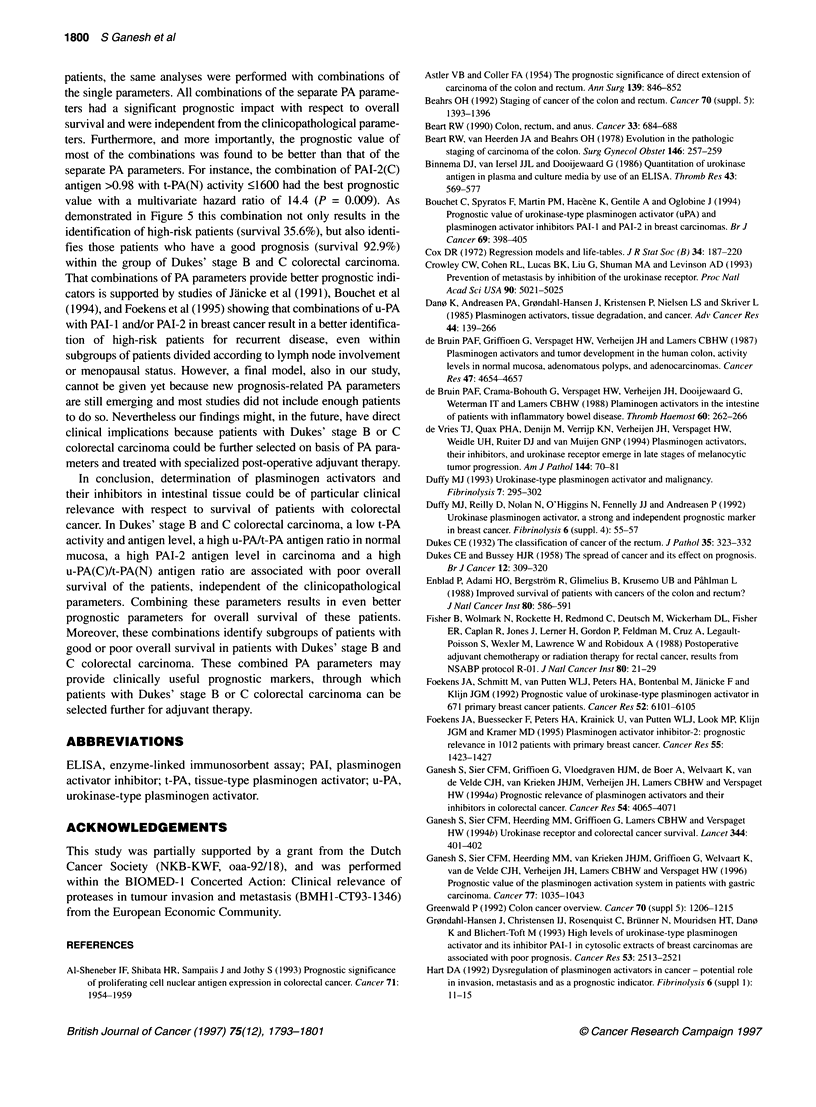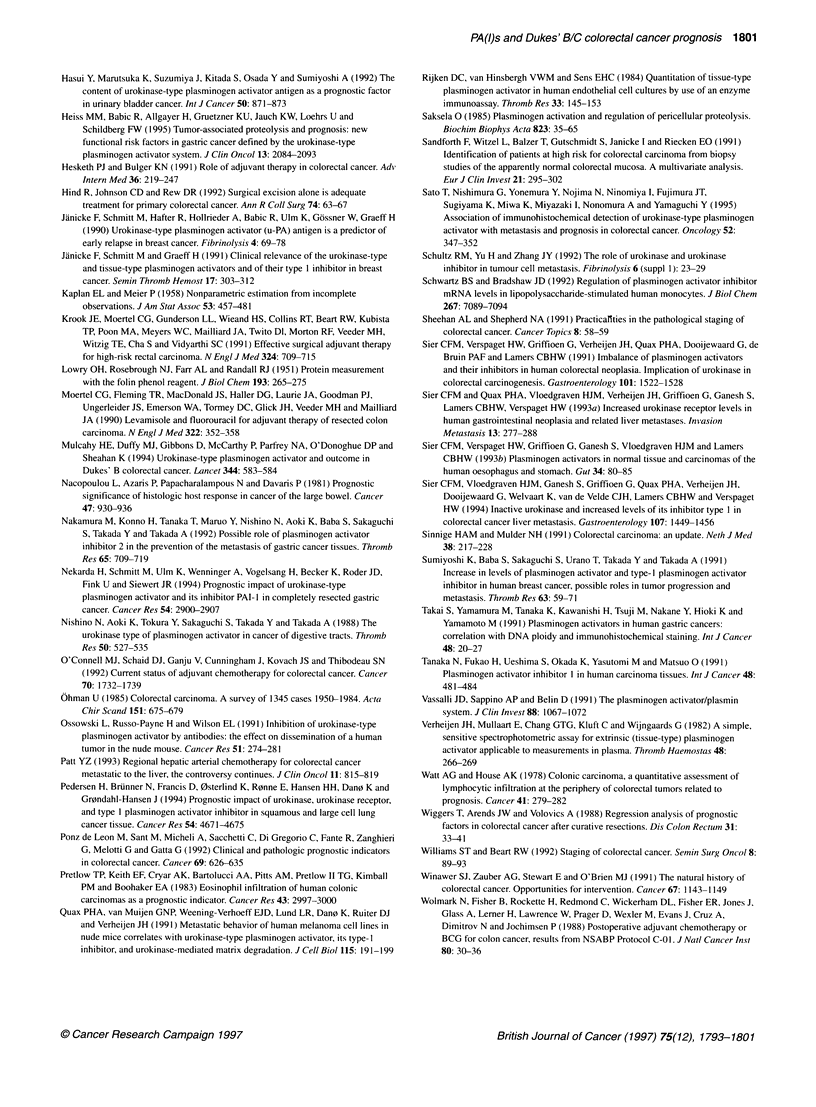# Contribution of plasminogen activators and their inhibitors to the survival prognosis of patients with Dukes' stage B and C colorectal cancer.

**DOI:** 10.1038/bjc.1997.306

**Published:** 1997

**Authors:** S. Ganesh, C. F. Sier, M. M. Heerding, J. H. van Krieken, G. Griffioen, K. Welvaart, C. J. van de Velde, J. H. Verheijen, C. B. Lamers, H. W. Verspaget

**Affiliations:** Department of Gastroenterology and Hepatology, University Hospital Leiden, The Netherlands.

## Abstract

Despite the advances in pre-, peri- and post-operative medical care of colorectal carcinoma patients, the prognosis has improved only marginally over recent decades. Thus, additional prognostic indicators would be of great clinical value to select patients for adjuvant therapy. In previous studies we found that colorectal carcinomas have a marked increase of the urokinase-type of plasminogen activator (u-PA), and the inhibitors PAI-1 and PAI-2, whereas the tissue-type plasminogen activator (t-PA) is found to be decreased in comparison with adjacent normal mucosa. In the present study we evaluated the prognostic value of several plasminogen activation parameters, determined in both normal and carcinomatous tissue from colorectal resection specimens, for overall survival of 136 Dukes' stage B and C colorectal cancer patients, in relation to major clinicopathological parameters. Uni- and multivariate analyses indicated that a high PAI-2 antigen level in carcinoma, a low t-PA activity and antigen level and a high u-PA/t-PA antigen ratio in adjacent normal mucosa are significantly associated with a poor overall survival. A high ratio of u-PA antigen in the carcinomas and t-PA antigen in normal mucosa, i.e. u-PA(C)/t-PA(N), was found to be predictive of a poor overall survival as well. All these parameters were found to be prognostically independent of the clinicopathological parameters. Multivariate analysis of combinations of these prognostically significant plasminogen activation parameters revealed that they are important independent prognostic indicators and have in fact a better prognostic value than their separate components. Based on these combined parameters, subgroups of patients with Dukes' stage B and C colorectal cancer could be identified as having either a high or a low risk regarding overall survival. In conclusion, these findings emphasize the relevance of the intestinal plasminogen activation system for survival prognosis of patients with colorectal cancer and, in the future, might constitute a patient selection criterion for adjuvant therapy.


British Journal of Cancer (1997) 75(12), 1793–1801
© 1997 Cancer Research Campaign

### Contribution of plasminogen activators and their inhibitors to the survival prognosis of patients with Dukes' stage B and C colorectal cancer

**S Ganesh[1], CFM Sier[1], MM Heerding[1], JHJM van Krieken[2], G Griffioen[1], K Welvaart[3], CJH van de Velde[3], JH Verheijen[4], CBHW Lamers[1] and HW Verspaget[1]**

Departments of [1]Gastroenterology and Hepatology; [2]Pathology and [3]Surgery, University Hospital Leiden; and [4]Gaubius Laboratory TNO-PG, Leiden, The Netherlands

**Summary** Despite the advances in pre-, peri- and post-operative medical care of colorectal carcinoma patients, the prognosis has improved only marginally over recent decades. Thus, additional prognostic indicators would be of great clinical value to select patients for adjuvant therapy. In previous studies we found that colorectal carcinomas have a marked increase of the urokinase-type of plasminogen activator (u-PA), and the inhibitors PAI-1 and PAI-2, whereas the tissue-type plasminogen activator (t-PA) is found to be decreased in comparison with adjacent normal mucosa. In the present study we evaluated the prognostic value of several plasminogen activation parameters, determined in both normal and carcinomatous tissue from colorectal resection specimens, for overall survival of 136 Dukes' stage B and C colorectal cancer patients, in relation to major clinicopathological parameters. Uni- and multivariate analyses indicated that a high PAI-2 antigen level in carcinoma, a low t-PA activity and antigen level and a high u-PA/t-PA antigen ratio in adjacent normal mucosa are significantly associated with a poor overall survival. A high ratio of u-PA antigen in the carcinomas and t-PA antigen in normal mucosa, i.e. u-PA(C)/t-PA(N), was found to be predictive of a poor overall survival as well. All these parameters were found to be prognostically independent of the clinicopathological parameters. Multivariate analysis of combinations of these prognostically significant plasminogen activation parameters revealed that they are important independent prognostic indicators and have in fact a better prognostic value than their separate components. Based on these combined parameters, subgroups of patients with Dukes' stage B and C colorectal cancer could be identified as having either a high or a low risk regarding overall survival. In conclusion, these findings emphasize the relevance of the intestinal plasminogen activation system for survival prognosis of patients with colorectal cancer and, in the future, might constitute a patient selection criterion for adjuvant therapy.

**Keywords:** colorectal cancer; plasminogen activator; prognosis; survival

Colorectal cancer is one of the leading causes of neoplastic morbidity and mortality in the Western world. Despite screening of high-risk individuals and advances in early diagnosis through the development of better diagnostic techniques, and better medical care and treatment, the prognosis of colorectal cancer has hardly changed over recent decades (Öhman, 1985; Enblad et al, 1988; Sinnige and Mulder, 1991; Winawer et al, 1991; Greenwald, 1992; Patt, 1993). To date, depth of tumour invasion, lymph node involvement and distant metastases, which form the basis of most classification systems, seem to be the most important intervention criteria and generally used prognostic factors for the survival of these patients (Beart et al, 1978; Beart, 1990; Sheenan and Shepherd, 1991; Beahrs, 1992; Williams and Beart, 1992). Primary treatment of patients with colorectal cancer is surgery. Although adjuvant therapy provided disappointing results for decades, recent studies offer some basis for optimism and changes in additional therapeutic regimens following surgery can be expected (Fisher et al, 1988; Wolmark et al, 1988; Moertel et al, 1990; Hesketh and Bulger, 1991; Krook et al, 1991; O'Connell et al, 1992). However, additional prognostic factors are necessary and may be of great importance for outcome prediction and treatment planning of subgroups of patients with colorectal cancer. In particular, patients with Dukes' stage B and C colorectal carcinoma still pose a major dilemma regarding the use of adjuvant therapy, as about 50% of these patients will be cured by surgery alone (Fisher et al, 1988; Wiggers et al, 1988; Wolmark et al, 1988; Hind et al, 1992). Therefore, it would be of great value to be able to identify by additional indicators patients within these histological subgroups with high or low risk for developing recurrent disease or who have a poor or good 5-year survival. Some of these individuals might then be selected for specialized adjuvant treatment.

Components of the plasminogen activation system play a role in the breakdown of the extracellular matrix and basement membranes. Consequently, they are important contributors to multifactorial processes such as proliferation, (tumour) cell migration, tumour invasion and subsequent metastasis formation (Danø et al, 1985; Saksela, 1985; Vassalli et al, 1991; Hart, 1992; Schultz et al, 1992; Duffy, 1993). Plasminogen activators (PAs) are proteolytic enzymes that catalyse the conversion of plasminogen to plasmin. Two distinct PA types are known: tissue-type PA (t-PA) and urokinase-type PA (u-PA). t-PA binds to fibrin and is therefore the major plasminogen activator involved in intravascular dissolution of blood clots. u-PA and its pro-form (pro-u-PA) bind to the surface of (cancer) cells via the urokinase receptor and are of

*Received 29 August 1996*
*Revised 2 December 1996*
*Accepted 12 December 1996*

*Correspondence to:* HW Verspaget, Department of Gastroenterology and Hepatology, University Hospital Leiden, Building 1, C4-P, PO Box 9600, 2300 RC Leiden, The Netherlands particular relevance in malignant processes. The activity of plasminogen activators is regulated by the plasminogen activator inhibitors PAI-1 and PAI-2. In vitro experiments with human carcinoma cell lines have shown that invasion and metastasis are correlated with enhanced expression of u-PA, and that these processes could be inhibited by antibodies against u-PA and by blocking of the u-PA receptor (Ossowski et al, 1991; Quax et al 1991; Crowley, 1993).

Many human tumour types have been found to have increased levels of u-PA, u-PA receptor, PAI-1 and/or PAI-2, and decreased levels of t-PA compared with their corresponding normal tissue counterparts (Sier et al, 1991; 1993*a*, b; Sumiyoshi et al, 1991; Tanaka et al, 1991; Nakamura et al, 1992; de Vries et al, 1994). These changes found in the plasminogen activation system were not only contributing to tumour invasion and metastasis but were also of particular clinical relevance because of their impact on prognosis. In particular, u-PA, PAI-1 and PAI-2 or combinations of these parameters have been found to be of prognostic relevance to overall and disease-free survival in breast (Jänicke et al, 1990; Duffy et al, 1992; Foekens et al, 1992; Grøndahl-Hansen et al, 1993; Bouchet et al, 1994), urinary bladder (Hasui et al, 1992), lung (Pedersen et al, 1994), gastric (Nekarda et al, 1994) and colorectal cancer (Ganesh et al, 1994*a*; Mulcahy et al, 1994). In colorectal cancer we recently found that even several plasminogen activation parameters in normal colorectal mucosa are of prognostic relevance for the overall survival of the patients (Ganesh et al, 1994*a*). Moreover, Mulcahy et al (1994) recently showed that high grades of epithelial cell u-PA staining in Dukes' B colorectal cancer is associated with a poorer 5-year survival than lower staining grades. Because of the prognostic impact of PA parameters and the clinical dilemma regarding adjuvant therapy of patients with colorectal cancer, we evaluated the possibility of identifying subgroups of these patients with differences in overall survival by determining the intestinal plasminogen activator and/or inhibitor levels. Several prognostically relevant clinical and histological parameters were determined of 136 patients operated for colorectal carcinoma lesions classified as Dukes' stage B or C. The prognostic relevance of the plasminogen activation parameters found in the colorectal carcinomas and their corresponding normal mucosa was compared with that of the clinicopathological parameters by performing uni- and multivariate survival analyses.

### MATERIALS AND METHODS

### Patients and study design

All the patients involved in this study were operated on for a histologically proven adenocarcinoma of the colorectum. The operations were performed from November 1983 to March 1988 at the Department of Surgery, University Hospital Leiden. Immediately after resection, fresh samples of carcinomas and adjacent normal mucosa, taken approximately 10 cm from the tumour, were frozen and stored at –70°C until extraction. From this group of patients several clinical and pathological data were evaluated and registered. The tumours were classified according to the Dukes' stage, as modified by Astler and Coller (Dukes, 1932; Astler and Coller, 1954; Beart et al, 1978), and 136 patients (60 women and 76 men, mean age 68.3 ± 0.9 years) with Dukes' B and C lesions, corresponding to UICC (1993) TNM stages I, II, and III, were included in the study. The pathological data of the carcinomas were revised by one pathologist (JvK), i.e. differentiation of grade into low, moderate or poor, and number of inflammatory cells and eosinophils into many, moderate or few. All patients entered the study at operation date and finished in the event of death ($n = 74$, 31 women and 43 men) or after a follow-up of at least 5 years at the common closing date ($n = 62$, 29 women and 33 men). After the primary resection or during follow-up, 18.3% (15/82) of the Dukes' B and 40.7% (22/54) of the Dukes' C patients were treated with either radiotherapy (6 and 16, respectively,), chemotherapy (three and one, respectively), or surgical resection of a locally recurrent adenocarcinoma or distant metastasis (three and one respectively), or a combination of these treatments (three and four respectively).

### Tissue extraction and protein concentration

Extracts were prepared from 50- to 100-mg wet tissue samples as described previously (de Bruin et al, 1987; Sier et al, 1991). In brief, the samples were homogenized in 1 ml of 0.1% (v/v) Tween 80; 0.1 M Tris-HCl buffer (pH 7.5), per 60 mg wet tissue at 0°C. The homogenates were centrifuged twice at 8000 ***g*** for 2.5 min, 4°C and the supernatant was stored at –70°C until analysis. Protein concentration of the supernatant was determined by the method of Lowry et al (1951).

### u-PA and t-PA antigen determination

The u-PA antigen determination was carried out using a sandwich ELISA according to Binnema et al (1986), with rabbit anti-u-PA as catching antibody. The samples were incubated overnight followed by affinopurified goat anti-u-PA IgG as second antibody. After washing, donkey anti-goat IgG conjugated with alkaline phosphatase was added and paranitrophenyl phosphate (1 mg $ml^{-1}$) was used as substrate. The amount of u-PA antigen in the samples was calculated from a nine-point standard curve of u-PA (0–3.3 ng $ml^{-1}$).

The t-PA antigen level was measured by an ELISA using goat anti-t-PA as catching antibody and anti-t-PA horseradish peroxidase conjugate as second antibody, according to Rijken et al (1984), whereas 3,3′;5,5′ tetramethylbenzidine was used as substrate. Quantities of t-PA antigen were calculated from an eight-point standard curve of t-PA (Biopool, Sweden, 0–32 ng $ml^{-1}$). Antigen concentrations were expressed finally as nanogram antigen per milligram protein (ng $mg^{-1}$ $protein^{-1}$).

### Assay for plasminogen activator activity u-PA and t-PA activities were measured enzymatically by a spectrophotometric assay (Verheijen et al, 1982). In brief, tissue extracts were incubated with plasminogen, fragments of fibrinogen and a chromogenic plasmin substrate, resulting in detection of total plasminogen activator activity. PA activities were distinguished by adding specific inhibitory antibodies against t-PA and u-PA (respectively rabbit anti-human t-PA IgG and goat anti-human u-PA IgM/IgD) to parallel incubations and the activity of the activators calculated by the amount of inhibition. The percentage (%) u-PA activity was calculated as 100 times the u-PA activity divided by the sum of the u-PA and t-PA activity. u-PA and t-PA standard preparations (National Institute of Biological Standards and Control, London, UK, batch nos 66/46 and 83/517 respectively) were included to express activities in international units. The inhibiting antibodies used were monospecific, showed

 © Cancer Research Campaign 1997

**Table 1** Levels of plasminogen activators and inhibitors in carcinomas and normal mucosa of patients with Dukes' stage B and C colorectal carcinomas

| | All patients (*n* = 134) | Dukes' B (*n* = 82) | Dukes' C (*n* = 52) | *P*-value |
|---|---|---|---|---|
| *Normal mucosa* | | | | |
| u-PA antigen[a] | 2.4 ± 0.1 | 2.3 ± 0.1 | 2.5 ± 0.2 | NS |
| u-PA activity[b] | 52.8 ± 3.1 | 53.0 ± 3.7 | 52.5 ± 5.5 | NS |
| %-PA activity[c] | 12.1 ± 0.7 | 12.6 ± 0.9 | 11.4 ± 1.0 | NS |
| t-PA antigen[a] | 4.9 ± 0.3 | 4.9 ± 0.4 | 4.8 ± 0.6 | NS |
| t-PA activity[b] | 1477 ± 74 | 1398 ± 81 | 1601 ± 141 | NS |
| u-PA/t-PA antigen ratio | 0.8 ± 0.1 | 0.8 ± 0.1 | 0.9 ± 0.1 | NS |
| *Carcinomas* | *n* = 136 | *n* = 82 | *n* = 54 | |
| u-PA antigen[a] | 14.0 ± 0.8 | 12.5 ± 0.7 | 16.2 ± 1.8 | NS |
| u-PA activity[b] | 97.8 ± 7.1 | 103.6 ± 9.3 | 89.0 ± 10.7 | NS |
| % u-PA activity[c] | 44.1 ± 1.8 | 45.7 ± 2.4 | 41.7 ± 2.9 | NS |
| t-PA antigen[a] | 2.2 ± 0.2 | 2.1 ± 0.2 | 2.2 ± 0.3 | NS |
| t-PA activity[b] | 494 ± 40 | 494 ± 50 | 493 ± 68 | NS |
| u-PA/t-PA antigen ratio | 11.4 ± 0.9 | 10.4 ± 1.0 | 13.0 ± 1.9 | NS |
| | *n* = 133 | *n* = 79 | *n* = 54 | |
| PAI-1 antigen[a] | 5.0 ± 0.8 | 4.3 ± 0.8 | 6.0 ± 1.6 | NS |
| | *n* = 131 | *n* = 77 | *n* = 54 | |
| PAI-2 antigen[a] | 3.3 ± 0.4 | 2.5 ± 0.3 | 4.5 ± 0.9 | 0.04 |
| | *n* = 133 | *n* = 82 | *n* = 51 | |
| u-PA(C)/u-PA(N) antigen ratio | 6.9 ± 0.4 | 6.6 ± 0.5 | 7.3 ± 0.8 | NS |
| | *n* = 134 | *n* = 82 | *n* = 52 | |
| u-PA(C)/t-PA(N) antigen ratio | 5.0 ± 0.4 | 4.6 ± 0.5 | 5.6 ± 0.8 | NS |

Mean ± s.e. [a]ng $mg^{-1}$ protein; [b]mIU $mg^{-1}$ protein; [c](100 × u-PA activity)/(u-PA activity + t-PA activity). C, carcinoma; N, normal mucosa.

**Table 2** Univariate analysis of clinicopathological parameters in relation to overall survival of patients with Dukes' stage B and C colorectal carcinomas

| Parameters | Number of survivors/total (%) | Median survival in months | Hazard ratio (95% CI, *P*-value) |
|---|---|---|---|
| Gender | | | |
| Female | 29/60 (48.3) | 62.9 | |
| Male | 33/76 (43.4) | 47.0 | 1.2 (0.8–1.9, NS) |
| Age (years) | | | |
| ≤ 66.1 | 34/54 (63.0) | > 83.0 | |
| > 66.1 | 28/82 (34.1) | 40.0 | 2.1 (1.3–3.6, 0.004) |
| Localization | | | |
| Right colon | 20/49 (40.8) | 51.5 | |
| Left colon | 42/87 (48.3) | 65.4 | 0.8 (0.5–1.3, NS) |
| Differentiation grade | | | |
| Moderate/well | 28/61 (45.9) | 62.2 | |
| Poor | 34/75 (45.3) | 53.5 | 1.0 (0.7–1.7, NS) |
| Diameter | | | |
| < 4 cm | 18/29 (62.1) | > 79.0 | |
| ≥ 4 cm | 44/107 (41.1) | 45.5 | 1.9 (1.0–3.5, NS) |
| Inflammatory cells | | | |
| Many | 19/31 (61.3) | 83.0 | |
| Moderate/few | 42/99 (42.4) | 46.8 | 1.9 (1.0–3.6, 0.04) |
| Eosinophils | | | |
| Many | 13/20 (65.0) | > 83.0 | |
| Moderate/few | 48/110 (43.6) | 51.0 | 2.0 (0.9–4.4, NS) |
| Dukes' stage | | | |
| Dukes' B | 45/82 (54.9) | > 87.0 | |
| Dukes' C | 17/54 (31.5) | 34.2 | 1.9 (1.2–3.1, 0.004) |

CI, confidence interval.

© Cancer Research Campaign 1997

**Figure 1** Overall survival curves according to Dukes' stage B and C of the patients with colorectal cancer. Values are the number of patients alive–dead at the end of follow-up. Univariate hazard ratio 1.9 (95% confidence interval 1.2–3.1)

no cross-reactivity and blocked maximum standard u-PA and t-PA completely. Activator activities were expressed finally as milli-international units u-PA or t-PA per milligram protein (mIU $mg^{-1}$ protein).

### PAI-1 and PAI-2 antigen determination

Total PAI-1 antigen, i.e. latent, active and complexed PAI-1, was determined using the Tintelize PAI-1 ELISA (Biopool, Umeå, Sweden) without prior denaturation of the samples as described previously (Sier et al, 1991). In brief, mouse monoclonal anti-human PAI-1 was used as catching antibody. After incubation with the tissue homogenates a goat polyclonal anti-human PAI-1, conjugated to peroxidase, was used to form a sandwich ELISA and orthophenylenediamine was added as substrate. The assay included the use of quenching and non-specific antibodies to exclude falsely elevated results. In order to increase the sensitivity of the assay sample volumes of up to 40 μl were used, instead of the recommended 20 μl, resulting in a detection limit of 0.3 ng $ml^{-1}$.

The determination of PAI-2 antigen was performed using the Tintelize PAI-2 ELISA from Biopool (Sier et al, 1991). The first antibody used was mouse monoclonal anti-human PAI-2 and the second was goat polyclonal anti-PAI-2 IgG conjugated to peroxidase. Orthophenylenediamine was added as substrate. Unspecific response was excluded using quenching antibodies. The detection limit was decreased to 0.5 ng $ml^{-1}$ by using 50 μl of homogenate instead of 20 μl and by increasing sample incubation, conjugate incubation and substrate incubation times. Antigen concentrations were expressed finally as nanogram antigen per milligram protein (ng $mg^{-1}$ protein).

**Table 3** Multivariate analysis of plasminogen activator parameters in colorectal carcinoma and corresponding normal mucosa of patients with Dukes' stage B and C lesions in relation to the overall survival of the patients

| Parameters | Number of survivors/total (%) | Median survival in months | Multivariate hazard ratio (95% CI, P-value) |
|---|---|---|---|
| *Normal mucosa* | | | |
| t-PA antigen[a] | | | |
| > 7.39 | 19/30 (63.3) | > 81.0 | |
| ≤ 7.39 | 41/104 (39.4) | 46.0 | 2.0 (1.0–4.2, 0.05) |
| t-PA activity[b] | | | |
| > 1600 | 30/50 (60.0) | > 83.0 | |
| ≤ 1600 | 30/84 (35.7) | 44.5 | 2.1 (1.2–3.8, 0.008) |
| u-PA/t-PA antigen ratio | | | |
| ≤ 0.22 | 17/24 (70.8) | > 81.0 | |
| > 0.22 | 43/110 (39.1) | 45.0 | 2.8 (1.2–6.6, 0.02) |
| *Carcinoma* | | | |
| u-PA/t-PA antigen ratio | | | |
| ≤ 3.91 | 24/38 (63.2) | > 83.0 | |
| > 3.91 | 38/98 (38.8) | 44.5 | 1.7 (0.9–3.2, NS) |
| PAI-2 antigen[a] | | | |
| ≤ 0.98 | 23/37 (62.2) | > 83.0 | |
| > 0.98 | 38/94 (40.4) | 44.5 | 1.9 (1.0–3.4, 0.05) |
| u-PA(C)/t-PA(N) antigen ratio | | | |
| ≤ 6.73 | 53/104 (51.0) | > 83.0 | |
| > 6.73 | 7/30 (23.3) | 26.0 | 2.9 (1.6–5.1, < 0.001) |

CI, confidence interval. [a]ng $mg^{-1}$ protein. [b]mIU $mg^{-1}$ protein. C, carcinoma; N, normal mucosa. Multivariate analysis was performed by adjusting the separate PA parameters to all clinicopathological parameters indicated in Table 2.

 © Cancer Research Campaign 1997

**Figure 2** Overall survival curves according to high (> 1600 mIU $mg^{-1}$ protein) and low (≤ 1600 mIU $mg^{-1}$ protein) t-PA activity in normal mucosa of patients with Dukes' stage B and C colorectal carcinoma. Values are the number of patients alive–dead at the end of follow-up. Univariate hazard ratio 1.9 (95% confidence interval 1.2–3.2)

**Figure 3** Overall survival curves according to high (>0.98 ng $mg^{-1}$ protein) and low (≤ 0.98 ng $mg^{-1}$ protein) PAI-2 antigen level of the carcinomas of the patients with Dukes' stage B and C colorectal carcinoma. Values are the number of patients alive–dead at the end of follow-up. Univariate hazard ratio 2.0 (95% confidence interval 1.1–3.6)

### Statistical analyses

For the statistical analyses, the clinicopathological parameters were dichotomized as follows: tumour localization in the colon in right-sided (from caecum to splenic flexure) vs left-sided (from splenic flexure up to and including the rectum), Dukes' stage in Dukes' B vs C, differentiation grade in moderate/well vs poor, diameters of the tumour in < 4 cm vs > 4 cm and the number of inflammatory cells and eosinophils in many vs moderate/few. The cut-off points of the age and PA parameters were determined by stepwise increasing the level until the point of best discrimination was found in the Cox proportional hazards model (Cox, 1972), i.e. the optimal dichotomization.

Differences in PA levels between normal tissue and carcinomas and between Dukes' B and C patients were statistically tested (two-sided) using the paired and unpaired Student's *t*-test, with separate variance estimation if the standard deviations were significantly different according to the *F*-test. With a selection of the clinicopathological parameters and plasminogen activation parameters in both normal mucosa and carcinoma, univariate survival analysis was performed with the Cox proportional hazards model, using the EGRET statistical package (SERC Seattle, WA, USA), resulting in identification of covariates that were significantly correlated with the overall survival. The multivariate survival analyses were performed using the Cox proportional hazards method by separately adding the significant plasminogen activator variables to the clinicopathological parameters in order to estimate their independent prognostic value in the overall survival. The prognostically significant plasminogen activator parameters were also analysed in combinations by both uni- and multivariate analysis. Overall survival curves were constructed by the method of Kaplan and Meier (1958). Statistical values of $P \leq 0.05$ were considered significant.

### RESULTS

In this study 136 patients with Dukes' stage B or C colorectal carcinoma who had an initially curative resection were included, most of them were male (55.9%), and the overall survival of the patients at the common closing date was 45.6%.

Several plasminogen activation-related parameters were evaluated in both carcinoma and adjacent normal colorectal mucosa of the resection specimens. The distribution of the PA levels was characterized by high levels of u-PA and low levels of t-PA in carcinomatous tissue compared with normal colorectal mucosa, as illustrated in Table 1. Comparing the plasminogen activator levels between Dukes' stages B and C revealed no differences in the plasminogen activator parameters except for PAI-2, which was found to be higher in Dukes'C tumours. Several clinical and histological parameters, which could possibly have a relation with the overall survival of the patients, were also evaluated. After optimal

© Cancer Research Campaign 1997

**Figure 4** Overall survival curves according to high (> 6.73) and low (≤ 6.73) antigen ratio of u-PA in carcinomas and t-PA in normal mucosa of the patients with Dukes' stage B and C colorectal carcinoma. Values are the number of patients alive–dead at the end of follow-up. Univariate hazard ratio 2.2 (95% confidence interval 1.3–3.5)

| | | | | |
|---|---|---|---|---|
| PAI-2 (C) | L | H | L | H |
| t-PA (N) act | H | H | L | L |
| Event | 1/14 | 18/35 | 13/21 | 38/59 |
| Hazard ratio | | 9.0 | 12.5 | 14.4 |
| *P*-value | | 0.03 | 0.02 | 0.009 |

**Figure 5** Graphic presentation of the percentage survival of subgroups of Dukes' stage B and C colorectal cancer patients according to combinations of high (H) or low (L) PAI-2 antigen level (cut-off 0.98 ng $mg^{-1}$ protein) in the carcinoma with a high or low level of t-PA activity (cut-off 1600 mIU $mg^{-1}$ protein) in the corresponding normal mucosa. Events indicate the number of patients dead over total at the end of follow-up. Hazard ratios and corresponding *P*-values were obtained by Cox's multivariate analysis. Dotted line indicates overall survival (45.6%) of all patients at the end of follow-up dichotomization of the parameters univariate analyses were performed, and higher age of the patients, relatively few inflammatory cells in the carcinomas and advanced Dukes' stage C were found to be prognostic for a poor survival at the study closing date (Table 2, Figure 1). Within the different histological stages we found a gradual decrease in the percentage of survival at the study closing date ($B_1$, 71.4%; $B_2$, 49.2%; $C_1$, 41.2% and $C_2$, 27.0%), which is in agreement with data from the literature. A large size, i.e. diameter, of the tumour and a low number of eosinophils tended to be associated with a poor survival. The overall survival of these patients was found to be independent of the other parameters such as gender and localization and differentiation grade of the tumour. Patients receiving additional treatment after the primary resection or during follow-up were found to have a significantly ($P < 0.01$) poorer overall survival than patients having only primary surgical intervention, 27.0% (10/37) vs 52.5% (52/99) respectively, with a similar pattern within the groups of patients having either Dukes' stage B or stage C colorectal cancer.

All the plasminogen activator parameters were also optimally dichotomized and in the univariate analyses we found that several of these parameters had a prognostic value for overall survival (Table 3). In normal mucosa a low level of t-PA activity (Figure 2) and antigen, a high level of u-PA/t-PA antigen ratio, and in carcinomas a high level of u-PA/t-PA antigen ratio and PAI-2 antigen (Figure 3) were significantly associated with a poor overall survival of the patients. Similarly, a high level of the u-PA(C)/t-PA(N) antigen ratio (Figure 4) was predictive of a poor survival. With all these univariately significant plasminogen activator parameters multivariate analyses were performed by adjusting each parameter separately to all the clinicopathological parameters (Table 3). Only the u-PA/t-PA antigen ratio in the carcinomas lost its significance whereas all other parameters, both in carcinoma and normal mucosa, were found to be prognostically independent of the clinicopathological parameters for the overall survival of the patients. Regrading these parameters, only age and Dukes' stage were found to be consistently associated with survival in the multivariate analyses.

Because several plasminogen activator parameters and the inhibitor PAI-2 were found to have an independent prognostic value for overall survival we also evaluated whether a better subdivision of patients with respect to survival could be made by combining these parameters and performing uni- and multivariate analyses. This is illustrated in Figure 5, which shows that patients with a low PAI-2 in the carcinomas and a high t-PA activity in the normal mucosa have a good overall survival (92.9%) as opposed to the patients with a high PAI-2 in the carcinomas and a low t-PA activity in the normal mucosa (survival 35.6%), whereas the other two combinations show an intermediate survival. Moreover, these combined parameters had a significant prognostic value independent of the clinicopathological parameters.

### DISCUSSION

The prognosis of a colorectal carcinoma patient is generally predicted by the well-established Dukes' classification, based on the depth of tumour invasion and metastasis (Dukes, 1932; Astler and Coller, 1954; Dukes and Bussey, 1958). It is known, however, that in the different Dukes' stages, particularly stage B and C, the

© Cancer Research Campaign 1997

prognosis of patients can vary considerably. The reason for this variation could be the fact that the Dukes' stage reflects a stage in the course of cancer growth rather than its biological behaviour (Beart et al, 1978; Beart, 1990; Sheehan and Shepherd, 1991; Beahrs, 1992; Williams and Beart, 1992; Patt, 1993). Although in recent years several major studies have been performed to evaluate adjuvant chemotherapy for colorectal cancer (Fisher et al, 1988; Wolmark et al, 1988; Moertel et al, 1990; Hesketh and Bulger, 1991; Krook et al, 1991; O'Connell et al, 1992), it remains of great relevance to evaluate other (biological) features for their prognostic impact and patient selection. This could be helpful in clinical decisions, and would possibly offer a better approach to adjuvant therapy planning. The role of plasminogen activators in neoplastic growth is well established by in vitro and clinical studies. The plasminogen activators and their inhibitors play a key role in the proteolytic cascade that is responsible for the breakdown of several components of the extracellular matrix, which surrounds the tumour cells. This process results in invasion and subsequently metastasis formation (Danø et al, 1985; Saksela, 1985; Vassalli et al, 1991; Ossowski et al, 1991; Quax et al, 1991; Hart, 1992; Schultz et al, 1992; Crowley et al, 1993; Duffy, 1993). We determined several components of the PA system in carcinomatous tissue and adjacent normal mucosa in a subgroup of 136 Dukes' stage B or C colorectal carcinoma patients, and evaluated their prognostic value for overall survival in comparison with several major clinicopathological parameters.

From the clinicopathological parameters, higher age of the patient (> 66.1 years), Dukes' stage C, and moderate to few inflammatory cells in the carcinoma were significantly associated with poor overall survival of the patients, whereas a diameter of the tumour ≥ 4 cm and the presence of relatively few eosinophils in the carcinoma tended to be associated. The observation that a severe intratumoral inflammatory reaction, mostly lymphocytic and eosinophilic infiltration, is associated with a better survival in colorectal cancer has also been reported by others (Watt and House, 1978; Nacopoulou et al, 1981; Pretlow et al, 1983; Ponz de Leon et al, 1992). The inflammatory response within these malignancies could be considered as an attempt of the host to enclose the carcinomatous tissue and to destroy cancer cells. However, its exact role in cancer prognosis in general remains to be established. In agreement with our previous study we did not find differentiation and localization of carcinomas within the large bowel to have an impact on survival (Ganesh et al, 1994*a*). Regarding the PA parameters we found that a high u-PA/t-PA antigen ratio and a high PAI-2 antigen level in carcinomas was associated with a poor overall survival, but only the latter remained significant in the multivariate analysis. In contrast to Mulcahy et al (1994) and Sato et al (1995), who recently reported a positive association between epithelial u-PA staining and the survival of colorectal cancer patients, we found no relation between survival and u-PA. The only association we did find recently in this context is a highly significant prognostic impact of the u-PA receptor level on colorectal cancer survival, also within Dukes' B and C (Ganesh et al, 1994*b*). The prognostic value of a high PAI-2 antigen level was found to be independent of the severity of the inflammatory reaction within the carcinoma. This finding was remarkable as there is some evidence that PAI-2 is involved in inflammatory processes (Schwartz and Bradshaw, 1992). Our study once more reveals that a low t-PA level (antigen and activity) and a high u-PA/t-PA antigen ratio in normal mucosa of patients with colorectal cancer, and a high u-PA(C)/t-PA(N) antigen ratio are significantly associated with poor overall survival of the patients. Thus, also in a subgroup of colorectal carcinoma patients, i.e. Dukes' stage B and C, several PA parameters in both carcinoma and adjacent normal mucosa seem to be of major prognostic value for overall survival. In recent years several studies have been reported that surveyed the relation between plasminogen activators and inhibitors in other human malignancies and the prognosis of the patients. Most of these studies have been carried out in breast cancer and show that high u-PA and PAI-1 antigen levels are associated with the survival of the patients, independent of other prognostic factors, including lymph node status (Jänicke et al, 1990; Duffy et al, 1992; Foekens et al, 1992; Grøndahl-Hansen et al, 1993; Bouchet et al, 1994). Because of the fact that these PA parameters could discern patients at high risk of developing recurrent disease or having a poor survival in the node-negative group, they already form the basis for selection of patients for further treatment with chemotherapy. Also, high levels of u-PA, PAI-1 and PAI-2 have been found in carcinomas of the stomach and oesophagus (Nishino et al, 1988; Takai et al, 1991; Tanaka et al, 1991; Nakamura et al, 1992; Sier et al, 1993*b*). Moreover, recent reports of Nekarda et al (1994), Heiss et al (1995), and our group (Ganesh et al, 1996) have shown that these parameters are of prognostic value for the survival of patients with gastric cancer, PAI-1 being an independent prognostic factor. Furthermore, a high u-PA antigen level in urinary bladder cancer (Hasui et al, 1992) and a high PAI-1 antigen level in pulmonary adenocarcinoma (Pedersen et al, 1994) have also been reported to be of prognostic value for the survival of patients. In contrast to these studies we did not find PAI-1 to be of prognostic significance, as opposed to PAI-2, which was found to be independently associated with overall survival of Dukes' stage B or C colorectal cancer patients. It is known that in colorectal cancer the presence of metastasis has more influence on prognosis than the rate of invasion. Therefore, it could be postulated that the PAI-2 antigen level of the carcinomas might indicate the metastatic potential of colorectal cancer because of its independent prognostic impact on survival of these patients. However, in breast cancer opposite findings have been reported, i.e. a high PAI-2 level was associated with a relatively good prognosis, particularly in patients with a high tumour u-PA or PAI-1 level. It was speculated that in breast tumours PAI-2 acts as a true inhibitor of u-PA activity (Bouchet et al, 1994; Foekens et al, 1995). The origin of the differences in the association of PAI-1 and PAI-2 with survival prognosis between breast and colorectal cancer is unclear and remains to be elucidated.

In almost all prognostic studies, the plasminogen activator parameters have been determined in carcinomatous tissue. In gastric and colorectal carcinoma patients, however, the t-PA level in normal mucosa seems also to be associated with survival (Ganesh et al, 1994*a*, 1996 and this study). This cannot be attributed to a cancer patient's specific mucosal plasminogen activator phenotype as the t-PA level in patients' colorectal mucosa was previously found to be identical to that of unaffected mucosa of inflammatory bowel disease patients (de Bruin et al, 1988). The fact that pathophysiological parameters of normal mucosa can be of prognostic value is supported by other studies. Increased cellular proliferation in normal mucosa of patients with colorectal cancer, for instance, has been found to be an independent prognostic variable for survival (Sandforth et al, 1991; Al-Sheneber et al, 1993). In this study, several PA parameters in both normal colorectal mucosa and carcinoma were found to be of prognostic relevance. However, to determine whether the separate PA parameters give maximal information regarding the prognostic value of the PA system in our

© Cancer Research Campaign 1997

patients, the same analyses were performed with combinations of the single parameters. All combinations of the separate PA parameters had a significant prognostic impact with respect to overall survival and were independent from the clinicopathological parameters. Furthermore, and more importantly, the prognostic value of most of the combinations was found to be better than that of the separate PA parameters. For instance, the combination of PAI-2(C) antigen >0.98 with t-PA(N) activity ≤1600 had the best prognostic value with a multivariate hazard ratio of 14.4 ($P$ = 0.009). As demonstrated in Figure 5 this combination not only results in the identification of high-risk patients (survival 35.6%), but also identifies those patients who have a good prognosis (survival 92.9%) within the group of Dukes' stage B and C colorectal carcinoma. That combinations of PA parameters provide better prognostic indicators is supported by studies of Jänicke et al (1991), Bouchet et al (1994), and Foekens et al (1995) showing that combinations of u-PA with PAI-1 and/or PAI-2 in breast cancer result in a better identification of high-risk patients for recurrent disease, even within subgroups of patients divided according to lymph node involvement or menopausal status. However, a final model, also in our study, cannot be given yet because new prognosis-related PA parameters are still emerging and most studies did not include enough patients to do so. Nevertheless our findings might, in the future, have direct clinical implications because patients with Dukes' stage B or C colorectal carcinoma could be further selected on basis of PA parameters and treated with specialized post-operative adjuvant therapy.

In conclusion, determination of plasminogen activators and their inhibitors in intestinal tissue could be of particular clinical relevance with respect to survival of patients with colorectal cancer. In Dukes' stage B and C colorectal carcinoma, a low t-PA activity and antigen level, a high u-PA/t-PA antigen ratio in normal mucosa, a high PAI-2 antigen level in carcinoma and a high u-PA(C)/t-PA(N) antigen ratio are associated with poor overall survival of the patients, independent of the clinicopathological parameters. Combining these parameters results in even better prognostic parameters for overall survival of these patients. Moreover, these combinations identify subgroups of patients with good or poor overall survival in patients with Dukes' stage B and C colorectal carcinoma. These combined PA parameters may provide clinically useful prognostic markers, through which patients with Dukes' stage B or C colorectal carcinoma can be selected further for adjuvant therapy.

### ABBREVIATIONS

ELISA, enzyme-linked immunosorbent assay; PAI, plasminogen activator inhibitor; t-PA, tissue-type plasminogen activator; u-PA, urokinase-type plasminogen activator.

### ACKNOWLEDGEMENTS

This study was partially supported by a grant from the Dutch Cancer Society (NKB-KWF, oaa-92/18), and was performed within the BIOMED-1 Concerted Action: Clinical relevance of proteases in tumour invasion and metastasis (BMH1-CT93-1346) from the European Economic Community.

### REFERENCES

Al-Sheneber IF, Shibata HR, Sampaiis J and Jothy S (1993) Prognostic significance of proliferating cell nuclear antigen expression in colorectal cancer. *Cancer* **71**: 1954–1959

Astler VB and Coller FA (1954) The prognostic significance of direct extension of carcinoma of the colon and rectum. *Ann Surg* **139**: 846–852

Beahrs OH (1992) Staging of cancer of the colon and rectum. *Cancer* **70** (suppl. 5): 1393–1396

Beart RW (1990) Colon, rectum, and anus. *Cancer* **33**: 684–688

Beart RW, van Heerden JA and Beahrs OH (1978) Evolution in the pathologic staging of carcinoma of the colon. *Surg Gynecol Obstet* **146**: 257–259

Binnema DJ, van Iersel JJL and Dooijewaard G (1986) Quantitation of urokinase antigen in plasma and culture media by use of an ELISA. *Thromb Res* **43**: 569–577

Bouchet C, Spyratos F, Martin PM, Hacène K, Gentile A and Oglobine J (1994) Prognostic value of urokinase-type plasminogen activator (uPA) and plasminogen activator inhibitors PAI-1 and PAI-2 in breast carcinomas. *Br J Cancer* **69**: 398–405

Cox DR (1972) Regression models and life-tables. *J R Stat Soc (B)* **34**: 187–220

Crowley CW, Cohen RL, Lucas BK, Liu G, Shuman MA and Levinson AD (1993) Prevention of metastasis by inhibition of the urokinase receptor. *Proc Natl Acad Sci USA* **90**: 5021–5025

Danø K, Andreasen PA, Grøndahl-Hansen J, Kristensen P, Nielsen LS and Skriver L (1985) Plasminogen activators, tissue degradation, and cancer. *Adv Cancer Res* **44**: 139–266

de Bruin PAF, Griffioen G, Verspaget HW, Verheijen JH and Lamers CBHW (1987) Plasminogen activators and tumor development in the human colon, activity levels in normal mucosa, adenomatous polyps, and adenocarcinomas. *Cancer Res* **47**: 4654–4657

de Bruin PAF, Crama-Bohouth G, Verspaget HW, Verheijen JH, Dooijewaard G, Weterman IT and Lamers CBHW (1988) Plaminogen activators in the intestine of patients with inflammatory bowel disease. *Thromb Haemost* **60**: 262–266

de Vries TJ, Quax PHA, Denijn M, Verrijp KN, Verheijen JH, Verspaget HW, Weidle UH, Ruiter DJ and van Muijen GNP (1994) Plasminogen activators, their inhibitors, and urokinase receptor emerge in late stages of melanocytic tumor progression. *Am J Pathol* **144**: 70–81

Duffy MJ (1993) Urokinase-type plasminogen activator and malignancy. *Fibrinolysis* **7**: 295–302

Duffy MJ, Reilly D, Nolan N, O'Higgins N, Fennelly JJ and Andreasen P (1992) Urokinase plasminogen activator, a strong and independent prognostic marker in breast cancer. *Fibrinolysis* **6** (suppl. 4): 55–57

Dukes CE (1932) The classification of cancer of the rectum. *J Pathol* **35**: 323–332

Dukes CE and Bussey HJR (1958) The spread of cancer and its effect on prognosis. *Br J Cancer* **12**: 309–320

Enblad P, Adami HO, Bergström R, Glimelius B, Krusemo UB and Påhlman L (1988) Improved survival of patients with cancers of the colon and rectum? *J Natl Cancer Inst* **80**: 586–591

Fisher B, Wolmark N, Rockette H, Redmond C, Deutsch M, Wickerham DL, Fisher ER, Caplan R, Jones J, Lerner H, Gordon P, Feldman M, Cruz A, Legault-Poisson S, Wexler M, Lawrence W and Robidoux A (1988) Postoperative adjuvant chemotherapy or radiation therapy for rectal cancer, results from NSABP protocol R-01. *J Natl Cancer Inst* **80**: 21–29

Foekens JA, Schmitt M, van Putten WLJ, Peters HA, Bontenbal M, Jänicke F and Klijn JGM (1992) Prognostic value of urokinase-type plasminogen activator in 671 primary breast cancer patients. *Cancer Res* **52**: 6101–6105

Foekens JA, Buessecker F, Peters HA, Krainick U, van Putten WLJ, Look MP, Klijn JGM and Kramer MD (1995) Plasminogen activator inhibitor-2: prognostic relevance in 1012 patients with primary breast cancer. *Cancer Res* **55**: 1423–1427

Ganesh S, Sier CFM, Griffioen G, Vloedgraven HJM, de Boer A, Welvaart K, van de Velde CJH, van Krieken JHJM, Verheijen JH, Lamers CBHW and Verspaget HW (1994*a*) Prognostic relevance of plasminogen activators and their inhibitors in colorectal cancer. *Cancer Res* **54**: 4065–4071

Ganesh S, Sier CFM, Heerding MM, Griffioen G, Lamers CBHW and Verspaget HW (1994*b*) Urokinase receptor and colorectal cancer survival. *Lancet* **344**: 401–402

Ganesh S, Sier CFM, Heerding MM, van Krieken JHJM, Griffioen G, Welvaart K, van de Velde CJH, Verheijen JH, Lamers CBHW and Verspaget HW (1996) Prognostic value of the plasminogen activation system in patients with gastric carcinoma. *Cancer* **77**: 1035–1043

Greenwald P (1992) Colon cancer overview. *Cancer* **70** (suppl 5): 1206–1215

Grøndahl-Hansen J, Christensen IJ, Rosenquist C, Brünner N, Mouridsen HT, Danø K and Blichert-Toft M (1993) High levels of urokinase-type plasminogen activator and its inhibitor PAI-1 in cytosolic extracts of breast carcinomas are associated with poor prognosis. *Cancer Res* **53**: 2513–2521

Hart DA (1992) Dysregulation of plasminogen activators in cancer – potential role in invasion, metastasis and as a prognostic indicator. *Fibrinolysis* **6** (suppl 1): 11–15

 © Cancer Research Campaign 1997

Hasui Y, Marutsuka K, Suzumiya J, Kitada S, Osada Y and Sumiyoshi A (1992) The content of urokinase-type plasminogen activator antigen as a prognostic factor in urinary bladder cancer. *Int J Cancer* **50**: 871–873

Heiss MM, Babic R, Allgayer H, Gruetzner KU, Jauch KW, Loehrs U and Schildberg FW (1995) Tumor-associated proteolysis and prognosis: new functional risk factors in gastric cancer defined by the urokinase-type plasminogen activator system. *J Clin Oncol* **13**: 2084–2093

Hesketh PJ and Bulger KN (1991) Role of adjuvant therapy in colorectal cancer. *Adv Intern Med* **36**: 219–247

Hind R, Johnson CD and Rew DR (1992) Surgical excision alone is adequate treatment for primary colorectal cancer. *Ann R Coll Surg* **74**: 63–67

Jänicke F, Schmitt M, Hafter R, Hollrieder A, Babic R, Ulm K, Gössner W, Graeff H (1990) Urokinase-type plasminogen activator (u-PA) antigen is a predictor of early relapse in breast cancer. *Fibrinolysis* **4**: 69–78

Jänicke F, Schmitt M and Graeff H (1991) Clinical relevance of the urokinase-type and tissue-type plasminogen activators and of their type 1 inhibitor in breast cancer. *Semin Thromb Hemost* **17**: 303–312

Kaplan EL and Meier P (1958) Nonparametric estimation from incomplete observations. *J Am Stat Assoc* **53**: 457–481

Krook JE, Moertel CG, Gunderson LL, Wieand HS, Collins RT, Beart RW, Kubista TP, Poon MA, Meyers WC, Mailliard JA, Twito DI, Morton RF, Veeder MH, Witzig TE, Cha S and Vidyarthi SC (1991) Effective surgical adjuvant therapy for high-risk rectal carcinoma. *N Engl J Med* **324**: 709–715

Lowry OH, Rosebrough NJ, Farr AL and Randall RJ (1951) Protein measurement with the folin phenol reagent. *J Biol Chem* **193**: 265–275

Moertel CG, Fleming TR, MacDonald JS, Haller DG, Laurie JA, Goodman PJ, Ungerleider JS, Emerson WA, Tormey DC, Glick JH, Veeder MH and Mailliard JA (1990) Levamisole and fluorouracil for adjuvant therapy of resected colon carcinoma. *N Engl J Med* **322**: 352–358

Mulcahy HE, Duffy MJ, Gibbons D, McCarthy P, Parfrey NA, O'Donoghue DP and Sheahan K (1994) Urokinase-type plasminogen activator and outcome in Dukes' B colorectal cancer. *Lancet* **344**: 583–584

Nacopoulou L, Azaris P, Papacharalampous N and Davaris P (1981) Prognostic significance of histologic host response in cancer of the large bowel. *Cancer* **47**: 930–936

Nakamura M, Konno H, Tanaka T, Maruo Y, Nishino N, Aoki K, Baba S, Sakaguchi S, Takada Y and Takada A (1992) Possible role of plasminogen activator inhibitor 2 in the prevention of the metastasis of gastric cancer tissues. *Thromb Res* **65**: 709–719

Nekarda H, Schmitt M, Ulm K, Wenninger A, Vogelsang H, Becker K, Roder JD, Fink U and Siewert JR (1994) Prognostic impact of urokinase-type plasminogen activator and its inhibitor PAI-1 in completely resected gastric cancer. *Cancer Res* **54**: 2900–2907

Nishino N, Aoki K, Tokura Y, Sakaguchi S, Takada Y and Takada A (1988) The urokinase type of plasminogen activator in cancer of digestive tracts. *Thromb Res* **50**: 527–535

O'Connell MJ, Schaid DJ, Ganju V, Cunningham J, Kovach JS and Thibodeau SN (1992) Current status of adjuvant chemotherapy for colorectal cancer. *Cancer* **70**: 1732–1739

Öhman U (1985) Colorectal carcinoma. A survey of 1345 cases 1950–1984. *Acta Chir Scand* **151**: 675–679

Ossowski L, Russo-Payne H and Wilson EL (1991) Inhibition of urokinase-type plasminogen activator by antibodies: the effect on dissemination of a human tumor in the nude mouse. *Cancer Res* **51**: 274–281

Patt YZ (1993) Regional hepatic arterial chemotherapy for colorectal cancer metastatic to the liver, the controversy continues. *J Clin Oncol* **11**: 815–819

Pedersen H, Brünner N, Francis D, Østerlind K, Rønne E, Hansen HH, Danø K and Grøndahl-Hansen J (1994) Prognostic impact of urokinase, urokinase receptor, and type 1 plasminogen activator inhibitor in squamous and large cell lung cancer tissue. *Cancer Res* **54**: 4671–4675

Ponz de Leon M, Sant M, Micheli A, Sacchetti C, Di Gregorio C, Fante R, Zanghieri G, Melotti G and Gatta G (1992) Clinical and pathologic prognostic indicators in colorectal cancer. *Cancer* **69**: 626–635

Pretlow TP, Keith EF, Cryar AK, Bartolucci AA, Pitts AM, Pretlow II TG, Kimball PM and Boohaker EA (1983) Eosinophil infiltration of human colonic carcinomas as a prognostic indicator. *Cancer Res* **43**: 2997–3000

Quax PHA, van Muijen GNP, Weening-Verhoeff EJD, Lund LR, Danø K, Ruiter DJ and Verheijen JH (1991) Metastatic behavior of human melanoma cell lines in nude mice correlates with urokinase-type plasminogen activator, its type-1 inhibitor, and urokinase-mediated matrix degradation. *J Cell Biol* **115**: 191–199

Rijken DC, van Hinsbergh VWM and Sens EHC (1984) Quantitation of tissue-type plasminogen activator in human endothelial cell cultures by use of an enzyme immunoassay. *Thromb Res* **33**: 145–153

Saksela O (1985) Plasminogen activation and regulation of pericellular proteolysis. *Biochim Biophys Acta* **823**: 35–65

Sandforth F, Witzel L, Balzer T, Gutschmidt S, Janicke I and Riecken EO (1991) Identification of patients at high risk for colorectal carcinoma from biopsy studies of the apparently normal colorectal mucosa. A multivariate analysis. *Eur J Clin Invest* **21**: 295–302

Sato T, Nishimura G, Yonemura Y, Nojima N, Ninomiya I, Fujimura JT, Sugiyama K, Miwa K, Miyazaki I, Nonomura A and Yamaguchi Y (1995) Association of immunohistochemical detection of urokinase-type plasminogen activator with metastasis and prognosis in colorectal cancer. *Oncology* **52**: 347–352

Schultz RM, Yu H and Zhang JY (1992) The role of urokinase and urokinase inhibitor in tumour cell metastasis. *Fibrinolysis* **6** (suppl 1): 23–29

Schwartz BS and Bradshaw JD (1992) Regulation of plasminogen activator inhibitor mRNA levels in lipopolysaccharide-stimulated human monocytes. *J Biol Chem* **267**: 7089–7094

Sheehan AL and Shepherd NA (1991) Practicalities in the pathological staging of colorectal cancer. *Cancer Topics* **8**: 58–59

Sier CFM, Verspaget HW, Griffioen G, Verheijen JH, Quax PHA, Dooijewaard G, de Bruin PAF and Lamers CBHW (1991) Imbalance of plasminogen activators and their inhibitors in human colorectal neoplasia. Implication of urokinase in colorectal carcinogenesis. *Gastroenterology* **101**: 1522–1528

Sier CFM and Quax PHA, Vloedgraven HJM, Verheijen JH, Griffioen G, Ganesh S, Lamers CBHW, Verspaget HW (1993*a*) Increased urokinase receptor levels in human gastrointestinal neoplasia and related liver metastases. *Invasion Metastasis* **13**: 277–288

Sier CFM, Verspaget HW, Griffioen G, Ganesh S, Vloedgraven HJM and Lamers CBHW (1993*b*) Plasminogen activators in normal tissue and carcinomas of the human oesophagus and stomach. *Gut* **34**: 80–85

Sier CFM, Vloedgraven HJM, Ganesh S, Griffioen G, Quax PHA, Verheijen JH, Dooijewaard G, Welvaart K, van de Velde CJH, Lamers CBHW and Verspaget HW (1994) Inactive urokinase and increased levels of its inhibitor type 1 in colorectal cancer liver metastasis. *Gastroenterology* **107**: 1449–1456

Sinnige HAM and Mulder NH (1991) Colorectal carcinoma: an update. *Neth J Med* **38**: 217–228

Sumiyoshi K, Baba S, Sakaguchi S, Urano T, Takada Y and Takada A (1991) Increase in levels of plasminogen activator and type-1 plasminogen activator inhibitor in human breast cancer, possible roles in tumor progression and metastasis. *Thromb Res* **63**: 59–71

Takai S, Yamamura M, Tanaka K, Kawanishi H, Tsuji M, Nakane Y, Hioki K and Yamamoto M (1991) Plasminogen activators in human gastric cancers: correlation with DNA ploidy and immunohistochemical staining. *Int J Cancer* **48**: 20–27

Tanaka N, Fukao H, Ueshima S, Okada K, Yasutomi M and Matsuo O (1991) Plasminogen activator inhibitor 1 in human carcinoma tissues. *Int J Cancer* **48**: 481–484

Vassalli JD, Sappino AP and Belin D (1991) The plasminogen activator/plasmin system. *J Clin Invest* **88**: 1067–1072

Verheijen JH, Mullaart E, Chang GTG, Kluft C and Wijngaards G (1982) A simple, sensitive spectrophotometric assay for extrinsic (tissue-type) plasminogen activator applicable to measurements in plasma. *Thromb Haemostas* **48**: 266–269

Watt AG and House AK (1978) Colonic carcinoma, a quantitative assessment of lymphocytic infiltration at the periphery of colorectal tumors related to prognosis. *Cancer* **41**: 279–282

Wiggers T, Arends JW and Volovics A (1988) Regression analysis of prognostic factors in colorectal cancer after curative resections. *Dis Colon Rectum* **31**: 33–41

Williams ST and Beart RW (1992) Staging of colorectal cancer. *Semin Surg Oncol* **8**: 89–93

Winawer SJ, Zauber AG, Stewart E and O'Brien MJ (1991) The natural history of colorectal cancer. Opportunities for intervention. *Cancer* **67**: 1143–1149

Wolmark N, Fisher B, Rockette H, Redmond C, Wickerham DL, Fisher ER, Jones J, Glass A, Lerner H, Lawrence W, Prager D, Wexler M, Evans J, Cruz A, Dimitrov N and Jochimsen P (1988) Postoperative adjuvant chemotherapy or BCG for colon cancer, results from NSABP Protocol C-01. *J Natl Cancer Inst* **80**: 30–36

© Cancer Research Campaign 1997